# Supplementary material for: The effect of nursing shared governance educational program on nurse managers’ knowledge for sustainable nursing excellence and empowerment
Source: PLoS One. 2025 Feb 11;20(2):e0307798. doi: 10.1371/journal.pone.0307798 (PMC11813110; doi:10.1371/journal.pone.0307798)
Supplement: Attachment — Submitted filename: point by point responses.docx [file pone.0307798.s001.docx]

5. Review Comments to the Author

Please use the space provided to explain your answers to the questions above. You may also include additional comments for the author, including concerns about dual publication, research ethics, or publication ethics. (Please upload your review as an attachment if it exceeds 20,000 characters)

Reviewer #1: Overall, I believe that the topic of shared governance is relevant to nurses and administrators around the world and that there are still many steps to take everywhere. Compliments therefore to the authors for taking up this topic in their environment. However, as to whether the main claim of this manuscript offers a significant contribution to the field of nursing management and organization of care, I have some reservations. My main reservation is that the conclusions of this study are rather logical which makes me doubt the scientific relevance. The goal of the intervention was increased awareness around shared governance and this was indeed achieved, measured immediately after the last meeting. Choosing awareness as an outcome variable of an educational intervention does not add much to the scientific discussions around nurse shared governance, after all, increases in knowledge and awareness about a topic immediately after an intervention is logical. An addition of longitudinal measurements (does awareness remain high over time) or change in behavior in practice (e.g. management style) would have added more value to the scientific discourse. The authors acknowledge this limitation already in the discussion section.

The title mentions that it is about female managers, however, the relevance of this explicit addition is not made clear in the manuscript. I recommend adjusting the title.

Author responses:

It was adjusted as follows: The Impact of Nursing Shared Governance Educational Program on Nurse Managers’ Knowledge for Sustainable Nursing Excellence and Empowerment.

- The reference style does not conform to PLOSONE's guidelines. Recommend to use a reference program.

Author responses:

The introduction was reviewed critically and the required modifications were done.

- A reporting checklist or guideline for conducting study and/or paper is missing.

Author responses:

Trend checklist was utilized and attached

- The introduction is long and not always succinctly described. For example, the first three sentences are wordy. It is not until sentence 4 that it becomes clear what this paper could be about. Advice is to critically review the introduction and write more concisely.

Author responses:

The introduction was reviewed critically and the required modifications were done.

- In lines 280-290 is referred to a previous study of the authors, however the reference is unclear. Precisely this information is interesting because after the introduction on shared governance, I as a reader am also particularly keen to read about the state of shared governance and nursing management and organizational structure in the research setting/country. This knowledge helps a reader to assess whether I could generalize the results of this study to my setting. Nor is this explained anywhere else in the paper.

Author responses:

The reference was clarified as per the valuable recommendations of the respected reviewer.

- The authors use self-designed questionnaires. About the 'professional nursing shared governance knowledge questionnaire', it is written that it was designed based on literature. There is no reference to which literature. For the 'evaluation' questionnaire it is not described on which basis it was designed. Both questionnaires are missing, no reference also to a supplementary file. With regard to validity, it was stated that face and content validity had been established, but information on which technique was used for this was lacking. Nothing was said about internal consistency or structural validity. This limits the opportunities for replication of the studies methodology for other researchers.

Author responses:

Thank you for the valuable and constructive feedback. We confirm that all the required modifications were done as per your valuable guidance and directions.

- The tables are clear in structure and design- The discussion is described in a lengthy manner. I advise the authors to improve readability by making the text more concise.

Author responses:

Thank you for the valuable and constructive feedback. We confirm that all the required modifications were done as per your valuable guidance and directions.

- The paper is about nurse managers, however in the discussion literature of both nurses and nurse managers is used interchangeably. The rationale for this is sometimes lacking. For example in line 636-641.Based on the intervention, outcome variable and study design, reflection on effect of training on the outcome variable 'awareness' would be of added value.

Author responses:

Thank you for the valuable and constructive feedback. We need to clarify that the reason for using nurses and nurse managers in the discussion literature is returning to the nature of the supporting studies that we considered to support our study main findings.

Reviewer #2: I would like to thank the authors for the opportunity to review this manuscript. The subject of the article is of interest to the community specialized in health and nursing. However, there are serious problems with formatting and compliance with the journal's standards (tables, citations, data availability) that have not been met. A revision in English is needed, perhaps with the help of a native speaker.

I am putting forward my suggestions point by point so that a version of greater quality and robustness can be presented.

Authors responses

Dear Respected Professor Reviewer,

Kindly we would like to thank you for the constructive and valuable review and we confirm that we followed all your valuable instructions and fixed it as per your valuable guidance.

Title: Could be adjusted to make it more objective and focused on the object of study. As it is not a gender study, why do the authors use the word "women"? There is no emphasis throughout the text.

Author responses:

It was adjusted as follows: The Impact of Nursing Shared Governance Educational Program on Nurse Managers’ Knowledge for Sustainable Nursing Excellence and Empowerment.

Introduction:I missed more current references on the subject, the last ones are from 2019. I suggest that you also add other existing interventions in the literature that have increased knowledge on the subject.It is necessary to review all citations according to the journal's guidelines. The model used is vancouver numeric.Line 289

Author responses:

Thank you for the valuable and constructive feedback. We confirm that all the required modifications were done as per your valuable guidance and directions.

- The citation of the study is not clear, there are only a few names (Farghaly, S. M., El-Bialy, G. G., & Dowidar).

Author responses:

Thank you for the valuable and constructive feedback. We confirm that all the required modifications were done as per your valuable guidance and directions. All references and its citation were reviewed and modified.

Lines 294 - 297. The use of the PICO strategy is interesting, but in this case there would be no comparator, as no analysis is carried out with professionals who are not part of the session. I suggest removing or suppressing the use of C.

Author responses:

Thank you for the valuable and constructive feedback. We confirm that all the required modifications were done as per your valuable guidance and directions.

Method:

Lines 313-316: Add more information about the institution, for example: number of beds, professionals and level of complexity.

Author responses:

Thank you for the valuable and constructive feedback. We confirm that all the required modifications were done as per your valuable guidance and directions.

Lines 321-324: Were all the nurse managers invited and did they agree to take part in the survey? Do you work with the population?

Author responses:

Thank you for the valuable and constructive feedback. It was explained as follows; The study's participants were chosen using a whole population sampling strategy (purposive sampling technique), and they included all first-line nurse managers who were employed by the hospital and available during the data collection period (N=50 out of 54), with the aim of assessing their understanding of the concept of professional nursing shared governance and their readiness to take part in the study. All first-line nurse managers working in the study setting met the inclusion criteria, while any staff nurse without a management nursing position did not.

Lines 328-354: It would be important for the instruments as well as the database to be added as supplementary documents for a better understanding of how the information was collected.

Author responses:

Thank you for the valuable and constructive feedback. We confirm that all the required modifications were done as per your valuable guidance and directions.

Lines 356-361: What were the Cronbach's alpha values? I suggest presenting them. And better detail the cut-off value for deciding consensus between the judges and how many rounds of consensus were necessary. How were these experts selected? Describe this section better.

Author responses:

Thank you for the valuable and constructive feedback. We confirm that all the required modifications were done as per your valuable guidance and directions.

Line 363: Change the title to description of the intervention.Table 1: Could contain more details of the intervention. For example: types of educational techniques, how many meetings, how long the sessions lasted and the number of participants in each (It's not clear whether all 50 managers took part in all the sessions).

Author responses:

Thank you for the valuable and constructive feedback. We confirm that all the required modifications were done as per your valuable guidance and directions. The required details about the intervention were added as per your valuable guidance and directions

Results:

Line 460: Exclude "predominantly female" wouldn't that be all female? after all there were no male participants in the study.The results could be written without dividing them into sections, so they don't flow and break the reader's train of thought.

Author responses:

Thank you for the valuable and constructive feedback. We confirm that all the required modifications were done as per your valuable guidance and directions.

Table 3 - I don't understand why the previous information question is important, since after the intervention it is logical for everyone to answer yes. In the variable "Sources of information" in the item "other sources", put in the table caption what the other sources are. Also in table 3, the satisfaction results are presented here and in table 5.It's not clear from the method, for example, because the instrument is not attached, how the complete response of concepts was assessed.

Author responses:

Thank you for the valuable and constructive feedback. We confirm that all the required modifications were done as per your valuable guidance and directions. We need to clarify that the question of the previous information was added for the reason of assessing their initial level of knowledge to assess the gap. In addition the overall satisfaction was removed from table 3 as it is presented in details and the required study tools are attached as per your valuable request.

Discussion:

The discussion needs more up-to-date references, as well as reflection on the authors' own studies already carried out at the same institution on the subject (https://www.sciedupress.com/journal/index.php/jnep/article/view/12157/7665, https://www.cureus.com/articles/24566-perception-of-shared-governance-among-registered-nurses-in-ambulatory-care-center-at-a-tertiary-care-hospital-in-saudi-arabia#!/).The discussion needs more in-depth reflections on the results found, as well as the authors' statements. I think that the limitations and implications for practice sections can be part of the discussion, and they are naturally arrived at without having to be divided up.

Author responses:

Thank you for the valuable and constructive feedback. We confirm that all the required modifications were done as per your valuable guidance and directions.

Conclusion:

Needs to be more concise and more focused on answering the objective.

Author responses:

Thank you for the valuable and constructive feedback. We confirm that all the required modifications were done as per your valuable guidance and directions.

Thank You
