# Supplementary material for: The effect of nursing shared governance educational program on nurse managers’ knowledge for sustainable nursing excellence and empowerment
Source: PLoS One. 2025 Feb 11;20(2):e0307798. doi: 10.1371/journal.pone.0307798 (PMC11813110; doi:10.1371/journal.pone.0307798)
Supplement: Attachment — Submitted filename: point_by_point_responses_auresp_2.docx [file pone.0307798.s002.docx]

Reviewer #3: Regards

Congratulations to the authors of the article.

The necessary reforms that the reviewers had proposed have been applied.

It would have been better to highlight the corrections in the revision of the article.

There is still little information about the validity and reliability of the questionnaire. Nothing was said about internal consistency or structural validity. Describe this section better. Because data collection tool, which is a questionnaire, more explanations are needed.

Author Responses:

Dear Respected professor reviewer,

Thank you for the constructive feedback and we confirm that the required modifications were done as following and it was highlighted in yellow within the manuscript:

Tool Adaptation

The study's instruments were created in English and then translated into Arabic using a forward-backward translation technique (Alyami et al. 2019). Five academic experts, whose areas of expertise included nursing administration and health governance, reviewed the tools for translation, face and content validity, and relevance. As a result, the questionnaire underwent little changes. To assess their validity and reliability, we used a variety of techniques. For example, we developed the coding instrument by applying Steps 1–6 of DeVellis' (2017) paradigm for scale construction (refer to Figure 1). Reviewing the literature study (10–12, 20–24) helped to clarify the construct and establish an item pool. Furthermore, Cronbach's alpha and content validity were used.

Validity of content

Five experts from the field, comprising two professors of nursing administration and three professors of health governance. The panel found typographical, punctuation, and word choice issues. In response to their recommendations, a few terms were modified. The knowledge questionnaire's stability over time was investigated in a pilot study with ten nurse supervisors to confirm the instruments' accuracy and functionality and to ascertain the time required to complete the questionnaires. The results revealed a high positive significant correlation (r ranged from 0.782 to 0.890). The pilot sample was absent from the study sample.

Internal consistency

In order to evaluate the instruments' internal consistency, Cronbach's alpha was employed. It is a gauge for how well a collection of items represents a particular concept or dimension. more numbers indicate more reliability and internal consistency. The range is 0 to 1. The professional nursing shared governance knowledge questionnaire had a Cronbach's alpha rating of 0.89. This outcome shows that the study instruments' internal consistency and dependability are satisfactory.

Reviewer #4: Despite the effort to adapt the article based on the recommendations previously issued (strengthening it from a scientific point of view and the originality) and relevance in approaching this subject in a geographical area, which is still little studied, the article maintains its main weakness, since the way in which it is approached does not add to the knowledge that already exists on the subject. Analyzing what someone knows after undergoing a training process and realizing that they know more is the logical outcome for a training process. Perhaps an investigation that reports these changes on the ground, comparing before and after the formative process, would be a great contribution to scientific evolution. In this sense, analyzing the conclusion of the study, without evaluating the impact that the training of professionals had in the field, it is not possible to infer that -"consecutively, result in a positive workplace".

Only evaluating the results in the evolution of dynamics in clinical services can contribute to evaluating the effectiveness and efficiency of these processes.

Author responses:

Dear Respected Professor Reviewer,

Thank you for the valuable and constructive feedback and we confirm that we modified the conclusion section within the abstract as we removed the following sentence: -"consecutively, result in a positive workplace". We modified the title from impact to effect. In addition, we clarified the limitation of our study in a section titled limitation of the study based on your valuable recommendations as following:

The study's sample was restricted to a single study setting and a single pre-and post-test, which limits how broadly the findings may be applied. Furthermore, the current results could contain subjectivity and response bias because they are based on self-reported data. Considering this limitation, the study's conclusions have practical ramifications for how online training and learning programs are designed, particularly with regard to assessment procedures. These constraints might be addressed by longer-term, multi-site experiments that include intervention and control groups and analyse the dynamics of clinical services to assess the efficacy and efficiency of the procedures.

Thank you
